# EEG-based epilepsy detection with graph correlation analysis

**DOI:** 10.3389/fmed.2025.1549491

**Published:** 2025-03-12

**Authors:** Chongrui Tian, Fengbin Zhang

**Affiliations:** ^1^School of Computer Science and Technology, Harbin University of Science and Technology, Harbin, China; ^2^School of Information Engineering, East University of Heilongjiang, Harbin, China

**Keywords:** electroencephalogram, graph neural networks, correlation analysis, anomaly detection, abnormal EEG channels detection

## Abstract

Recognizing epilepsy through neurophysiological signals, such as the electroencephalogram (EEG), could provide a reliable method for epilepsy detection. Existing methods primarily extract effective features by capturing the time-frequency relationships of EEG signals but overlook the correlations between EEG signals. Intuitively, certain channel signals exhibit weaker correlations with other channels compared to the normal state. Based on this insight, we propose an EEG-based epilepsy detection method with graph correlation analysis (EEG-GCA), by detecting abnormal channels and segments based on the analysis of inter-channel correlations. Specifically, we employ a graph neural network (GNN) with weight sharing to capture target channel information and aggregate information from neighboring channels. Subsequently, Kullback-Leibler (KL) divergence regularization is used to align the distributions of target channel information and neighbor channel information. Finally, in the testing phase, anomalies in channels and segments are detected by measuring the correlation between the two views. The proposed method is the only one in the field that does not require access to seizure data during the training phase. It introduces a new state-of-the-art method in the field and outperforms all relevant supervised methods. Experimental results have shown that EEG-GCA can indeed accurately estimate epilepsy detection.

## 1 Introduction

The field of affective computing has witnessed significant development, drawing attention to emotion detection, especially in medical research related to epilepsy (1). While epilepsy, as a neurological disorder, manifests symptoms that encompass seizures, it often intertwines with fluctuations in emotional states. These emotional variations, a common symptom in epilepsy patients, are crucial for accurate disease monitoring and treatment (2).

Scalp electroencephalogram (EEG) stands as the primary tool for detecting seizures, capturing voltage changes between electrodes and providing spatial-temporal insights into brain activity (3–5). However, the current approach to seizure detection in EEGs relies on manual examination by experienced EEG readers, demanding substantial time and effort. Furthermore, discrepancies in diagnostic results may emerge due to varying opinions among experts (6).

To address these challenges, there is a pressing need for the development of automated and objective methods for epileptic seizure detection. While many studies have proposed deep learning (DL)-based models for automated seizure detection, several challenges persist (7–9). These models often train in a supervised approach, necessitating labeled seizure data that is both scarce and labor-intensive to obtain in real-world applications. Additionally, existing models frequently apply deep convolutional neural networks (CNNs) directly to time-series signals or spectrograms, overlooking crucial information related to physical distance-based and functional-based connectivity between different brain regions (10).

Recent studies have introduced graph learning techniques to capture relationships between EEG electrodes (i.e., EEG nodes) (6, 11, 12). However, these approaches fall short in considering local patterns, such as local sub-graphs and sub-structures, when learning EEG graphs. The inclusion of such local information could prove effective in detecting anomalies in EEG graphs, as demonstrated in other network analysis applications. In real-world applications, an imbalance in data availability between seizure and normal classes is common. Graph-based methods addressing this issue often employ graph augmentation, but not every augmentation technique is effective in EEG graphs (10), as some may compromise underlying brain region connectivities. Therefore, identifying appropriate augmentation strategies in EEG graphs that preserve semantic information is crucial for accurate seizure detection and localization (13).

This study delves into detecting the anomaly channels of EEG signal in patients with epilepsy (14). We propose an innovative method for epilepsy detection that distinctively focuses on exploring the inter-channel relationships within EEG signals, deemed essential for understanding the patient signal variations. We introduce an anomaly detection approach for EEG channels and segments based on inter-channel correlation analysis. This method utilizes Graph Neural Networks (GNNs) (15, 16) to capture the correlation between different channels, providing a more accurate reflection of anomaly changes. To achieve precise detection of anomaly channels in an EEG signal, we propose an EEG-based epilepsy detection method with graph correlation analysis (EEG-GCA), employing a weight-sharing GNN and aligning different channel information distributions with Kullback-Leibler (KL) (17) divergence regularization. During the testing phase, we detect anomalous channels and segments by measuring the correlation between two views, thereby achieving sensitive identification of abnormalities in epilepsy. Notably, our proposed method not only performs well in experiments but is also the only training approach that does not require access to seizure data. This research holds practical significance in improving the effectiveness of epilepsy patient treatment.

- We proposed a method named EEG-GCA for inter-channel correlation analysis simulating the correlation between channels in EEG, revealing subtle differences in patient anomaly changes. This algorithm provides a new approach to EEG signal processing.
- We redefined the anomaly channel detection of EEG as the correlation between channel feature distribution and their neighbors' distribution, and we designed an Unsupervised model to verify the effectiveness.
- The performance evaluation of the proposed abnormal EEG node and region detection is conducted on the extensive and comprehensive EEG seizure dataset TUSZ. The results demonstrate that EEG-GCA sets a new benchmark, achieving state-of-the-art performance on this dataset.

## 2 Related works

### 2.1 EEG analysis

Electroencephalogram analysis has become one of the prominent directions (18, 19). The following is a review of relevant work in this field, focusing on the application of different methods and technologies.

**(a)** Early approaches to epilepsy recognition primarily relied on traditional feature extraction techniques combined with machine learning algorithms (20, 21). Researchers extracted features from different domains, including time-domain, frequency-domain, and time-frequency-domain features, such as power spectral density and energy, to capture epilepsy-related patterns from EEG signals (22). Common machine learning models used in these early approaches included support vector machines (SVM) and decision trees (23, 24). While these methods achieved some success, their performance was often limited by the challenges of manually extracting relevant features and their inability to fully capture the complex dynamics of EEG signals.

**(b)** In recent years, deep learning methods have gained significant attention for their ability to enhance EEG-based epilepsy recognition (25). Architectures such as convolutional neural networks (CNNs) (26, 27) and recurrent neural networks (RNNs) (28) have been successfully applied, allowing models to learn feature representations in an end-to-end fashion. These deep learning techniques excel at capturing abstract and complex features from the raw EEG signals, significantly improving the accuracy of epilepsy recognition (24, 29). Furthermore, techniques such as transfer learning and multimodal fusion have been extensively explored to improve the generalization capabilities of these models, enabling better performance on unseen data.

**(c)** Beyond EEG signals, there has been growing interest in integrating data from multiple modalities for epilepsy recognition tasks, including physiological signals, speech, and images (30). Cross-modal research aims to combine information from diverse sources, thereby enhancing the robustness and comprehensiveness of epilepsy detection systems (31, 32). This approach leverages complementary data to improve model performance, offering a more holistic view of the patient's condition and enhancing the reliability of diagnosis (33).

### 2.2 Canonical correlation analysis

Canonical correlation analysis (CCA) (34, 35) is a method that aims to find the linear transformation for measuring the relationship between two vectors. Give two vectors **X**_1_ and **X**_2_, the correlation $\rho =\frac{{\text{a}}^{T}\Sigma_{{\text{X}}_{1}{\text{X}}_{2}}\text{b}}{\sqrt{{\text{a}}^{T}\Sigma_{{\text{X}}_{1}{\text{X}}_{1}}\text{a}}\sqrt{{\text{b}}^{T}\Sigma_{{\text{X}}_{2}{\text{X}}_{2}}\text{b}}}$ is maximized by optimizing the objective :


(1) $$\begin{array}{l} \underset{\text{a},\text{b}}{\max}{\text{a}}^{T}\Sigma_{{\text{X}}_{1}{\text{X}}_{2}}\text{b},s.t.{\text{a}}^{T}\Sigma_{{\text{X}}_{1}{\text{X}}_{1}}\text{a}={\text{b}}^{T}\Sigma_{{\text{X}}_{2}{\text{X}}_{2}}\text{b}=\text{I} \end{array}$$

Soft-CCA (36) considers the decorrelation constraint as a term of loss and optimizes it jointly with other terms, and the objective of Soft CCA is:


(2) $$\begin{array}{l} \begin{array}{c} \underset{\theta 1,\theta 2}{\max}Tr\left(P_{\theta_{1}}^{T}\left({\text{X}}_{1}\right)P_{\theta_{2}}\left({\text{X}}_{2}\right)\right)\\ s.t.P_{\theta_{1}}^{T}\left({\text{X}}_{1}\right)P_{\theta_{1}}\left({\text{X}}_{1}\right)=P_{\theta_{2}}^{T}\left({\text{X}}_{2}\right)P_{\theta_{2}}\left({\text{X}}_{2}\right)=\text{I} \end{array} \end{array}$$

where **I** is the identity matrix, and Equation 2 can be rewritten as:


(3) $$\begin{array}{l} \begin{array}{c} \underset{\theta 1,\theta 2}{\min}\|P_{\theta_{1}}\left({\text{X}}_{1}\right)-P_{\theta_{2}}\left({\text{X}}_{2}\right){\|}_{F}^{2}+\\ \lambda \left(L_{SDL}\left(P_{\theta_{1}}\left({\text{X}}_{1}\right)\right)+L_{SDL}\left(P_{\theta_{2}}\left({\text{X}}_{2}\right)\right)\right) \end{array} \end{array}$$

where *P*_θ_1__ and *P*_θ_2__ are the neural networks used to learn the representations of the two views. $\|P_{\theta_{1}}\left({\text{X}}_{1}\right)-P_{\theta_{2}}\left({\text{X}}_{2}\right){\|}_{F}^{2}$ is used to maximize the correlation between the two views, and $L_{SDL}$ is used to minimize the distance between *P*_θ_*i*__(**X**_*i*_) and the identity matrix.

### 2.3 Graph learning methods

Graph data, being non-Euclidean, poses a challenge for traditional convolution methods. The effective learning of information from graph data is an actively researched problem (37). In the context of graph data, the learned representation of nodes should encapsulate both the structural information of the graph and the attributes associated with each node. Existing graph learning methods can be broadly categorized as follows:

Truncated Random Walk-Based Methods: These methods operate on the assumption that nodes with similar network structures should have similar vector representations. A notable approach in this category is DeepWalk (38), which employs random walks to generate training data and leverages Word2vec (39) to learn node representations. Node2vec (40) captures homogeneity and structural equivalence through weighted random walks.

Methods Based on k-Order Distance Between Nodes in the Graph: These approaches, exemplified by methods like LINE (41) and GraRep (42), learn node representations by capturing k-order relational structure information, aiming to achieve high-quality node embeddings.

Deep Learning-Based Methods: Distinguished by their use of deep learning, these methods (43, 44) leverage the advantages of deep neural networks to extract high-order nonlinear relationships from graph data.

Graph neural networks (GNNs) (45) represent a significant advancement as they directly operate on graph data, aggregating each node's features with those of its neighbors. Building on GNNs, certain methods (46, 47) utilize GNNs to learn node representations. They employ adversarial learning to regularize these representations and predict the likelihood of an edge existing between a pair of nodes. However, these approaches predominantly rely on graph structure information.

Moreover, methods based on dual-autoencoders, such as AnomalyDAE (45) and Dual-SVDAE (48), use Graph Convolutional Networks to capture graph structure information. They combine this with multi-layer perceptrons (MLPs) to capture node attribute information, thereby making full use of attribute network information.

## 3 Method

**Figure 1:**
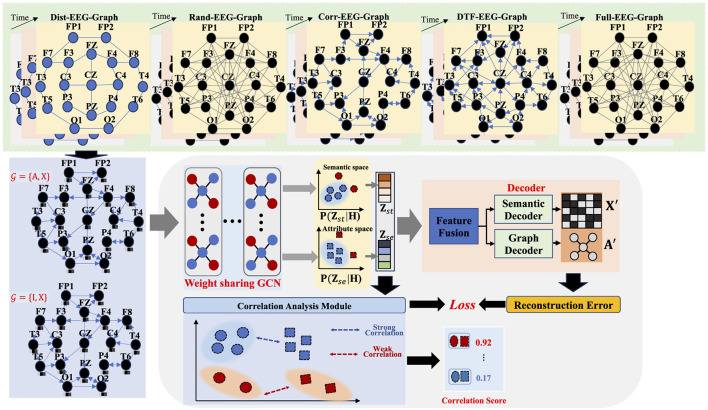
The framework of the proposed EGG-GCA.

In this section, we detail the EEG-GCA in Figure 1. It consists of a graph construct module, the information mining model, and a correlation analysis module. At first, we construct the EEG graph as input for our model. Then, we introduce an identity graph that represents the identity matrix, signifying no relationships between the channels. This graph aims to capture the features of each channel in the EEG data. Then, we input the EEG graph and identity graph into a weight-sharing GCN to learn the distribution of structural information and distribution of semantic information and pull the distributions to the same prior distribution through the Kullback-Leibler (KL) divergence. Finally, we sample the network structure embedding and node embedding from the learned distribution and maximize the correlation of normal nodes on the network structure distribution and node attribute distribution by using the CCA-based objective. The correlation score is used to detect the anomaly channels.

### 3.1 EEG graph construction

In this paper, we first construct the EEG graph as input. The EEG graph can be defined as an attributed network G={A,X}. Where **A** ∈ ℝ^*N*×*N*^ is the adjacency matrix that denotes the connection between each electrode. **X** ∈ ℝ^*N*×*D*^ denotes the feature matrix. **X**_*i*_ is feature of the *i*-th channel. Similar to the study, given an EEG clip, we construct five types of EEG graphs (12).

- *Dist* − *EEG* − *Graph* strives to embed the structure of electrode locations in the graph's adjacency matrix by leveraging the Euclidean distance between electrodes. Given that electrode locations remain fixed within an EEG recording cap, the same adjacency matrix is applied to all EEG clips. More precisely, the elements *a*_*ij*_ of the Dist-EEG-Graph are computed as follows:
(4) $$\begin{array}{l} a_{ij}=\{\begin{array}{cc} \text{exp}\left(-\frac{\left\|v_{i}-v_{j}\right\|}{\tau^{2}}\right),\; & if\;\|v_{i}-v_{j}|{|}^{2}\leq k,\\ 0,\; & if\;O.W. \end{array} \end{array}$$
  Here, ||•||represents the *l*_2_-norm, and τ is a scaling constant. The proximity between two electrodes, *v*_*i*_ and *v*_*j*_, is reflected by the proximity of *a*_*ij*_ to 1. In this paper, *k* is uniformly set to 0.9 across all EEG clips. Assigning a value of 0 to *a*_*ij*_ for distant electrodes introduces sparsity to the graph.
- *Corr* − *EEG* − *Graph* The purpose of this graph is to capture the functional connectivity between electrodes, which is encoded in the elements of the adjacency matrix defined as follows:
(5) $$\begin{array}{l} a_{ij}=\{\begin{array}{cc} \frac{corr\left({\text{X}}_{i},{\text{X}}_{j}\right)}{\left\|{\text{X}}_{i}\|\|{\text{X}}_{j}\right\|},\; & if\;v_{j}\in N\left(v_{i}\right),\\ 0,\; & if\;O.W. \end{array} \end{array}$$
  where *corr*(•) denotes the cross-correlation function, and *v*_*i*_ represents the top-3 neighborhood nodes of *v*_*i*_ with the highest normalized correlation. $N\left(v_{i}\right)$ is set to the top-3 neighborhood nodes to avoid overly connected graphs. Additionally, we only keep the top three edges for each node to prevent excessively connected graphs.
- *Rand* − *EEG* − *Graph* The construction of this graph is grounded on the assumption that all electrodes are interconnected and equally contribute to brain activities. The realization of this graph involves the formation of an adjacency matrix according to the following procedure:
(6) $$\begin{array}{l} a_{ij}=\{\begin{array}{cc} 0.5,\; & if\;i\neq j,\\ 1,\; & if\;O.W. \end{array} \end{array}$$
- *Full* − *EEG* − *Graph* Similar to the *Rand* − *EEG* − *Graph*, The construction of this graph is grounded on the assumption that all electrodes are interconnected and equally contribute to brain activities. But the *a*_*ij*_ is set as 1 for each connection.
- *DTF* − *EEG* − *Graph* The Directed Transfer Function Graph aims to represent the mutual influence between EEG channels, thereby modeling the functional connectivity of different brain regions. The adjacency matrix for this graph is defined as follows:
(7) $$\begin{array}{l} a_{ij}=\{\begin{array}{ll} \frac{corr(X_{i},X_{j})}{\sqrt{\sum_{m=1,m\neq i,j}^{n}}||corr(X_{i},X_{m}))|{|}^{2}}, & if{\text{ v}}_{\text{j}}\in N({\text{v}}_{\text{i}}),\\ 0, & if\text{ O}.\text{W}. \end{array} \end{array}$$

### 3.2 Weight-sharing GCN

To learn the correlation within the weight-sharing Graph Convolutional Network (GCN) for capturing the semantic and structural information of each node, we introduced an identity graph denoted as $G^{\prime }=\left\{\text{I},\text{X}\right\}$, where **I** represents the identity matrix signifying no relationships between the channels. This approach enhances the similarity between the semantic information and the graph structure information of each node by transferring the learned semantic information to all node features. Consequently, each channel feature can be obtained by inputting the identity graph into the Weight-Sharing GCN.

The construct EEG-graph G explicitly expresses the correlations between the channels in the EEG data, therefore, to capture the relationship information (structural information) between different channels, we input the EEG graph G={A,X} to the weight-sharing GCN.


(8) $$\begin{array}{l} \begin{array}{c} GCN\left(\text{X},\text{A}|W\right)=\varphi \left({\left(\text{D}\right)}^{-\frac{1}{2}}\text{A}{\left(\text{D}\right)}^{-\frac{1}{2}}\text{X}W\right) \end{array} \end{array}$$

where ***W*** is the learnable sharing weight, φ is activation function, and **D** is the diagonal degree matrix of the constructed EEG graph G.

To extract each channel information (node semantic information), the identity aggregation is designed which inputs the identity graph $G^{\prime }=\left\{\text{I},\text{X}\right\}$ to the weight-sharing GCN:


(9) $$\begin{array}{l} \begin{array}{c} GCN\left(\text{X},\text{I}|W\right)=\varphi \left(\text{I}\text{X}W\right) \end{array} \end{array}$$

### 3.3 Distribution alignment

After obtain the node structural information embedding **Z**_*st*_ and the node semantic information embedding **Z**_*se*_, we capture the structural distribution *q*(**Z**_*st*_|**X**, **A**) and semantic distribution *q*(**Z**_*se*_|**X**, **I**) for each node by Equation 10, respectively.


(10) $$\begin{array}{l} q\left(\text{Z}|\text{X},\text{A}\right)=\overset{N}{\underset{i=0}{\prod }}q\left({\text{z}}_{i}|\text{X},\text{A}\right) \end{array}$$

(11) $$\begin{array}{l} q\left({\text{z}}_{i}|\text{X},\text{A}\right)=N\left({\text{z}}_{i}|\mu_{i},\text{diag}\left(\sigma^{2}\right)\right) \end{array}$$

where **Z** is the embedding sampled from the distribution. **μ** is the mean vector and **σ** is the variance vector, which is learned by two different GCN layers.


(12) $$\begin{array}{l} \mu ={\text{GCN}}_{\mu }\left(\text{H},\text{A}|W\right) \end{array}$$

(13) $$\begin{array}{l} \sigma ={\text{GCN}}_{\sigma }\left(\text{H},\text{A}|W\right) \end{array}$$

where **μ**_*h*_ and **σ**_*h*_ denote the mean and variance vectors of the structural distribution learned by Equations 12, 13. Similarly, **μ**_*f*_ and **σ**_*f*_ are the mean and variance vectors of semantic distribution learned by Equations 12, 13.

To capture the correlation between the two distributions, we should align the structural distribution and semantic distribution. Due to it being harder to directly align two distributions, we use a Gaussian distribution as prior distribution *p* and use Kullback-Leibler (KL) divergence to align the two distributions wanting this prior distribution to achieve the desired effect.


(14) $$\begin{array}{l} L_{kl}=-\text{KL}\left[q\left({\text{Z}}_{st}|\text{X},\text{A}\right)\|p\left({\text{Z}}_{st}\right)\right]-\text{KL}\left[q\left({\text{Z}}_{se}|\text{X},\text{I}\right)\|p\left({\text{Z}}_{se}\right)\right] \end{array}$$

### 3.4 Decoder

The reconstruction of graph data is divided into two main parts, the reconstruction of the network structure and the reconstruction of the node attributes. Since nodes in graph data often have complex interactions with each other, it is necessary to fuse the features of each node with those of their neighbors.


(15) $$\begin{array}{l} {\text{Z}}_{f}={\text{Z}}_{st}+{\text{Z}}_{se} \end{array}$$

Then we use an *L*-layers Multi-Layer Perceptron (MLP) to reconstruct the node attributes.


(16) $$\begin{array}{l} {\text{Z}}_{f}^{\left(l\right)}=\sigma \left({\text{Z}}_{f}^{\left(l-1\right)}{\text{W}}^{\left(l-1\right)}+{\text{b}}^{\left(l-1\right)}\right) \end{array}$$

where ${\text{Z}}_{f}^{\left(l-1\right)}$, **Z**^(*l*)^, **W**^(*l*−1)^ and **b**^(*l*−1)^ are the input, output, the trainable weight and bias matrix of (*l* − 1)-th layer respectively, *l* ∈ {1, 2, ..., *L*}. σ(•) is the activation function. Finally, the reconstruction of node attributes $\hat{\text{X}}={\text{Z}}_{f}^{\left(L\right)}$ is obtained from the output of the last layer in MLP.

For the reconstruction of the network structure, we use an inner production of fusion embedding **Z**_*f*_ to reconstruct the network structure.


(17) $$\begin{array}{l} \hat{\text{A}}={\text{Z}}_{f}{\text{Z}}_{f}^{T} \end{array}$$

The reconstruction loss is defined as:


(18) $$\begin{array}{l} L_{dec}=\|\text{X}-\hat{\text{X}}\|+\|\text{A}-\hat{\text{A}}\| \end{array}$$

### 3.5 Correlation analysis objective

The objective of correlation analysis is to discern the relationship between structural distribution and semantic distribution. Initially, we sample the embeddings of structural information, denoted as **Z***st*, and semantic information, denoted as **Z***se*, from the distributions of structural features *q*(**Z***st*|**X**, **A**) and semantic features *q*(**Z***se*|**X**, **I**). Subsequently, we normalize the node embeddings for the two perspectives using the following procedure.


(19) $$\begin{array}{l} \begin{array}{c} {\text{Z}}_{st}^{\prime }=\frac{{\text{Z}}_{st}-\mu \left({\text{Z}}_{st}\right)}{\sigma \left({\text{Z}}_{st}\right)*N^{\frac{1}{2}}}\\ {\text{Z}}_{se}^{\prime }=\frac{{\text{Z}}_{se}-\mu \left({\text{Z}}_{se}\right)}{\sigma \left({\text{Z}}_{se}\right)*N^{\frac{1}{2}}} \end{array} \end{array}$$

Subsequently, as per the formulation in Equation 3, EEG-GCA enhances the correlation between the distributions of the two views by minimizing the invariance between the network structure embedding **Z**_*st*_ and the node attribute embedding **Z***f*. The invariance loss, denoted as Linv, is defined as:


(20) $$\begin{array}{l} L_{inv}=\|{\text{Z}}_{st}^{\prime }-{\text{Z}}_{se}^{\prime }{\|}_{F}^{2} \end{array}$$

To prevent collapsed solutions, we introduce the decorrelation loss, denoted as $L_{dco}$, which aims to guarantee that the individual dimensions of the features are uncorrelated.


(21) $$\begin{array}{l} {ℒ}_{dco}=||Z_{st}^{\prime T}Z_{st}^{\prime }-I|{|}_{F}^{2}+||Z_{se}^{\prime T}Z_{se}^{\prime }-I|{|}_{F}^{2} \end{array}$$

The CCA-based objective is defined as follows:


(22) $$\begin{array}{l} L_{CCA}=L_{inv}+\lambda *L_{dco} \end{array}$$

where λ is the trade-off between the two terms.

### 3.6 Loss function and anomaly score

The training objective of the proposed model involves optimizing the CCA-based loss along with minimizing the Kullback-Leibler (KL) divergence between the network structure distribution and the node attribute distribution.


(23) $$\begin{array}{l} L=L_{CCA}+L_{KL}+L_{dec} \end{array}$$

The anomaly score is defined as the correlation between channels with their structure information.

## 4 Performance evaluation

### 4.1 Dataset

In this study, we employed the Temple University Hospital EEG Seizure Corpus (TUSZ) v1.5.2 (12) as the benchmark dataset. This dataset stands out due to its extensive inclusion of seizure categories and patient samples, making it the dataset with the highest level of variability. Recorded over several years and by different generations of equipment, the dataset covers subjects of all ages, adding to its complexity and rendering it the most challenging for seizure detection. The EEG signals in TUSZ are captured using 19 channels based on the standard EEG 1,020 system. Table 1 provides an overview of the TUSZ dataset utilized in our experiments.

**Table 1:** Train and test sets of TUSZ used in the supervised method and unsupervised method.

**Data**	**Patients (% SZ)**	**EEG files (% SZ)**	**EEG clips (% SZ)**
*Train*_*Sup*_	591 (34.0%)	4,599 (18.9%)	38,613 (9.3%)
*Train*_*ours*_	493 (0%)	4,028 (0%)	35,019 (0%)
*Test*	45 (77.8%)	900 (25.6%)	8,848 (14.7%)

The percentages of the seizure data (SZ) is indicated in parenthesis.

During the training phase, we employed an equal number of normal clips as other supervised methods, omitting any seizure clips. In the testing phase, we utilized an equivalent number of test clips, encompassing both seizure and normal clips, for comparison against other supervised methods and our proposed approach. To assess the model's proficiency in seizure localization, we leveraged available annotations that specify focal and generalized seizure types from 23 distinct patients. It's noteworthy that, in epilepsy patients, focal and generalized seizure types are more prevalent compared to other seizure types, making them particularly relevant for our evaluation.

### 4.2 Baselines

We conducted a comprehensive evaluation of our proposed EEG-GCA method by comparing it with two distinct streams of deep learning-based approaches (12). The first stream involves well-established DL models operating in the EEG time-series and spectrograms domain, including EEGNet, EEG-TL, Dense-CNN, LSTM, and CNN-LSTM. The second stream focuses on DL models specifically designed for processing EEG graph data. Notably, our method differs from the others as it is deliberately trained without utilizing any seizure data in the training phase, ensuring a fair comparison. In addition, we compared another method, EEG-CGS (12), a graph-based method, which utilizes the constructed EEG graph and self-supervised learning to capture local structural and contextual information embedded in EEG graphs and detects the anomaly by designed anomaly scores.

In this paper, we explore six variations of EEG-GCA based on different input graph types: EEG_*d*_-GCA, EEG_*r*_-GCA, EEG_*c*_-GCA, EEG_*f*_-GCA, EEG_*t*_-GCA, and EEG_*l*_-GCA. These variations utilize Dist-EEG-Graph, Rand-EEG-Graph, Corr-EEG-Graph, Full-EEG-Graph, DTF-EEG-Graph, and Identity-EEG-Graph as their respective inputs. All methods were evaluated on the same dataset, with the comparative analysis focusing on assessing the robustness and generalization capabilities of EEG-GCA, particularly in scenarios where seizure data is limited or unavailable. To evaluate the performance of the models, we used three metrics: Area Under the Curve (AUC), Average Precision (AP), and Specificity (SPC). These metrics provide insights into the models' ability to distinguish between different classes, their precision in detecting positive samples, and their ability to correctly identify negative samples, respectively.

### 4.3 Detection of seizure clips and channels

The performance of the seizure clip detection experiment across various comparison methods is shown in Table 2. Among the supervised methods, Corr-DCRNN exhibits the highest accuracy of 0.4482, suggesting that it effectively utilizes correlation information between different EEG channels. This is a crucial feature for seizure detection, as it allows the model to capture temporal dependencies and spatial relationships within the EEG signal. However, despite its relatively high accuracy, the model still struggles with achieving high specificity, which is essential for minimizing false positives in seizure detection.

**Table 2:** Seizure clips detection result.

**Method**	**Acc**	**Precision**	**Spec**	**Method**	**Acc**	**Precision**	**Spec**
**Supervised**				**Unsupervised**			
EEGNet	0.4742	0.298	0.9021	EEG_*d*_-CGS	0.3076	0.3076	0.9450
EEG-TL	0.4001	0.2675	NA	EEG_*r*_-CGS	0.4285	0.3333	0.9291
Dense-CNN	0.4143	0.2746	0.8692	EEG_*c*_-CGS	0.2857	0.2857	0.9132
LSTM	0.3652	0.2635	0.8143	EEG_*f*_-CGS	0.2857	0.2857	0.9211
CNN-LSTM	0.3304	0.2572	0.8574	EEG_*t*_-CGS	0.3076	0.3076	0.9009
Dist-DCRNN	0.3414	0.2612	0.9321	–	–	–	–
Corr-DCRNN	0.4482	0.2711	0.9003	–	–	–	–
**Ours**							
EEG_*d*_-GCA	0.6812	0.3469	0.9714	EEG_*r*_-GCA	0.6636	0.3438	0.9429
EEG_*c*_-GCA	0.6847	0.3469	0.9714	EEG_*f*_-GCA	0.6832	0.3469	0.9714
EEG_*t*_-GCA	0.6848	0.3469	0.9714	EEG_*l*_-GCA	0.6625	0.3438	0.9429

In the unsupervised methods, EEG_*r*_-CGS, based on random graphs, performs the best with an accuracy of 0.4285. This result indicates that even without the use of labeled data, the model is still able to leverage the underlying structure in the EEG data to some extent. However, the performance gap between EEG_*r*_-CGS and supervised methods suggests that unsupervised learning still faces challenges in achieving comparable detection accuracy, particularly when it comes to fine-tuning the decision boundaries between seizure and non-seizure clips.

When comparing our proposed methods–EEG_*d*_-GCA, EEG_*r*_-GCA, EEG_*c*_-GCA, EEG_*f*_-GCA, EEG_*t*_-GCA, and EEG_*l*_-GCA–it is evident that the introduction of the Graph Correlation Attention (GCA) mechanism leads to significant improvements in both accuracy and specificity. The accuracy of our methods consistently outperforms both the supervised and unsupervised methods, with EEG_*d*_-GCA achieving the highest accuracy at 0.6812. This result is particularly noteworthy considering that EEG_*d*_-GCA utilizes the Dist-EEG-Graph as input, which focuses on capturing the structural relationships between different EEG channels. The combination of attention mechanisms with graph-based representations allows the model to selectively focus on the most informative features, leading to a more robust and accurate detection of seizure clips.

Interestingly, while EEG_*d*_-GCA achieves the highest accuracy, the other GCA variations (EEG_*r*_-GCA, EEG_*c*_-GCA, EEG_*f*_-GCA, EEG_*t*_-GCA, EEG_*l*_-GCA) also show consistently high performance with accuracy values close to 0.6847. This suggests that the robustness of the GCA mechanism is not highly sensitive to the specific graph input type, which makes these methods versatile across different graph representations of the EEG data. The consistently high specificity of around 0.9714 across all EEG-GCA methods indicates their effectiveness in minimizing false positives, which is a critical factor in the practical application of seizure detection systems.

### 4.4 Detection of synthetic anomalous channels

In this section, we focus on evaluating the performance of the proposed method EEG-GCA in reliably detecting anomalous channels. To this end, we generate a synthetic test set using normal clips from the training phase. Specifically, we average every 35 normal clips without overlap and then introduce anomalies into the averaged clips with a 3% probability. The anomalies are injected with a 0.03% probability, and at most one node is corrupted per averaged clip. The corruptions are applied both structurally and contextually. The structural corruption involves connecting the selected node to all other nodes in the average clip, while the contextual corruption alters the attribute vector of the node by replacing its feature vector with that of the node in the clip that has the largest Euclidean distance. After introducing these anomalies, we input the averaged clips, some of which contain anomalies, into the EEG-GCA networks that were trained on pure normal clips. The trained system then computes the anomaly scores for all channels.

The experimental results, as summarized in Table 3, demonstrate the effectiveness of our approach in the domain of anomaly detection. Our method outperforms both supervised and other unsupervised learning techniques across key evaluation metrics such as AUC, AP, and Specificity. Specifically, EEGNet, a supervised learning method, achieves a moderate performance with an AUC of 0.6182. However, it faces challenges when handling imbalanced datasets, which is a critical issue in real-world anomaly detection tasks. In contrast, EEG-GCA demonstrates remarkable improvements in AUC, with EEG_*r*_-GCA and EEG_*l*_-GCA achieving 0.9229 and 0.9238, respectively, highlighting the effectiveness of unsupervised learning techniques in addressing imbalances in the dataset. For AP, EEG-GCA surpasses the performance of the other methods. For instance, EEG_*l*_-GCA reaches an AP of 0.4792, significantly outperforming the supervised approaches. This indicates that our method is highly capable of accurately identifying anomalous events, which is crucial in real-world anomaly detection tasks such as sentiment recognition. Notably, EEG-GCA also excels in terms of Specificity, a metric that measures the ability to correctly identify normal samples and minimize false positives. Both EEG_*r*_-GCA and EEG_*l*_-GCA achieve Specificity values of 0.9722, outperforming all supervised models. This is particularly important as it demonstrates that our method can maintain high sensitivity while effectively reducing false positives, thereby improving the robustness and reliability of anomaly detection.

**Table 3:** Synthetic anomalous channels detection results.

**Type**	**Method**	**AUC**	**AP**	**Spec**
Supervised	EEGNet	0.6182	0.298	0.902
	EEG-TL	0.5913	0.2675	NA
	Dense-CNN	0.5877	0.2746	0.869
	LSTM	0.5198	0.2635	0.814
	CNN-LSTM	0.5412	0.2572	0.857
	Dist-DCRNN	05683	0.2612	0.932
	Corr-DCRNN	0.6122	0.2711	0.900
Unsupervised	EEG_*d*_-CGS	0.6182	0.0845	0.9455
	EEG_*r*_-CGS	0.8173	0.2675	0.9555
	EEG_*c*_-CGS	0.8241	0.2887	0.9555
	EEG_*f*_-CGS	0.8143	0.2960	0.9555
	EEG_*t*_-CGS	0.8241	0.2887	0.9555
Ours	EEG_*d*_-GCA	0.8903	0.4193	0.9667
	EEG_*r*_-GCA	0.9229	0.4618	0.9722
	EEG_*c*_-GCA	0.916	0.402	0.97
	EEG_*f*_-GCA	0.908	0.4325	0.9689
	EEG_*t*_-GCA	0.9101	0.4172	0.9678
	EEG_*l*_-GCA	0.9238	0.4792	0.9722

### 4.5 Ablation study

In the ablation study for seizure clip detection on synthetic anomalous channels, we explored two distinct approaches: Without Correlation and EEG-GCA. The results of this ablation analysis are summarized in Table 4.

**Table 4:** Ablation study on seizure clips detection results.

**Method**	**Without correlation**			**Ours**		
	**AUC**	**AP**	**Spec**	**AUC**	**AP**	**Spec**
EEG_*d*_-GCA	0.7579	0.3378	0.9644	0.8903	0.4193	0.9667
EEG_*r*_-GCA	0.8377	0.3431	0.9622	0.9229	0.4618	0.9722
EEG_*c*_-GCA	0.8513	0.4227	0.9678	0.9160	0.4020	0.9700
EEG_*f*_-GCA	0.8628	0.4025	0.9612	0.9080	0.4325	0.9689
EEG_*t*_-GCA	0.8355	0.3489	0.9533	0.9101	0.4172	0.9678
EEG_*l*_-GCA	0.8331	0.3301	0.9622	0.9238	0.4792	0.9722

In the Correlation approach, several graph construction methods were employed. Among these, EEG_*c*_-GCA emerged as the top performer, achieving the highest AUC (0.8513) and AP (0.4227), underscoring its effectiveness in seizure detection. This result emphasizes the importance of incorporating correlation in the graph construction process for improving detection accuracy. Notably, EEG_*r*_-GCA and EEG_*t*_-GCA also displayed competitive results, highlighting their resilience to the absence of correlation while still maintaining reasonable performance. These findings suggest that, even without explicit correlation, the models are capable of leveraging other aspects of the data for meaningful detection.

### 4.6 Visualization of EEG signal

To evaluate the abnormal channels in the electroencephalogram (EEG) segments during epileptic seizures, we visualize the seizure channel for generalized seizures.

**Figure 2:**
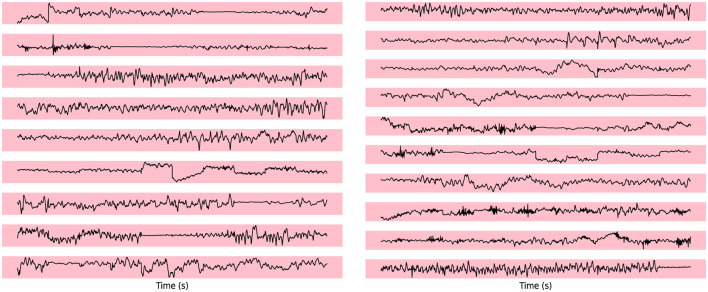
The visualization of seizure channel detection for generalized seizures.

In Figure 2, which represents a case of generalized seizures, our method demonstrates a high level of accuracy in detecting all abnormal channels. This robust performance aligns with our expectations for identifying anomalies during generalized seizure events, highlighting the reliability of our approach in such scenarios. The elevated anomaly scores observed in the seizure-affected channels provide strong evidence of the discriminatory power of our model, successfully distinguishing pathological EEG patterns from normal, baseline activity. This underscores the potential of our approach for real-time, accurate seizure detection.

## 5 Conclusion

In this paper, we introduce EEG-GCA, an unsupervised graph-based model designed for EEG-based epilepsy detection. The core of the methodology is centered around computing the correlation between individual EEG channels and their neighboring channels. The process begins with the construction of a graph representation of the EEG data, which enables the exploration of correlation patterns across the channels. A weight-sharing Graph Convolutional Network is then employed to effectively capture both the semantic and structural relationships among the channels. By aligning these distributions with a prior distribution, EEG-GCA learns the underlying correlations within the EEG data. The final stage involves detecting anomalous channels based on the correlation scores, with weak correlation scores indicating potential anomalies that may signify seizures. The experimental results demonstrate that EEG-GCA outperforms existing methods, achieving superior accuracy in detecting anomalous channels. This underscores the effectiveness of leveraging graph-based correlation techniques for the detection of epilepsy in EEG signals. In the future, we exploration involves integrating multi-modal data, such as incorporating additional physiological signals or patient-specific features, to further enhance the robustness and adaptability of models.

## Data Availability

The original contributions presented in the study are included in the article/supplementary material, further inquiries can be directed to the corresponding author.
